# Tumor-associated Tn and STn antigens: from molecular mechanism to precision diagnosis and treatment

**DOI:** 10.3389/fimmu.2025.1660852

**Published:** 2025-11-26

**Authors:** Shuailong Zhao, Cegang Fu, Boya Gong, Hongyan Wu, Ruitao Zhang, Huili Cai, Haidan Chen

**Affiliations:** 1 Department of Spinal Surgery, The First College of Clinical Medical Science, China Three Gorges University & Yichang Central People’s Hospital, Yichang, Hubei, China; 2 Department of Orthopedics, Haikou Orthopedic and Diabetes Hospital, Haikou Orthopedic and Diabetes Hospital of Shanghai Sixth People’s Hospital, Haikou, Hainan, China; 3 The First College of Clinical Medical Science, China Three Gorges University & Yichang Central People’s Hospital, Yichang, Hubei, China; 4 College of Basic Medicine, Three Gorges University, Yichang, Hubei, China; 5 Department of Anatomy, Medicine College, China Three Gorges University, Yichang, Hubei, China; 6 Department of Hematology, The First College of Clinical Medical Science, China Three Gorges University & Yichang Central People’s Hospital, Yichang, Hubei, China; 7 Hubei Provincial Clinical Research Center for Elderly Osteoporotic Fractures, Hubei, China

**Keywords:** tumor-associated glycan antigens (TACAs), Tn antigen, STn antigen, tumor immunity microenvironment (TIME), macrophage galactose-type lectin (MGL/CD301), Siglec, monoclonal antibody

## Abstract

**Background:**

Abnormal protein glycosylation is a key feature of tumors. Among the modifications, Tn antigen (GalNAcα1-Ser/Thr) and its sialylated derivative, STn antigen (Neu5Acα2-6GalNAcα1-O-Ser/Thr), are prominent tumor-associated carbohydrate antigens. These antigens exhibit abnormal accumulation in epithelial malignancies, including colorectal cancer, breast cancer, and pancreatic cancer. Their pathological overexpression primarily stems from inactivation of the COSMC/T-synthase axis, either due to genetic mutations or epigenetic silencing, leading to truncated O-glycan biosynthesis.

**Findings:**

Tn/STn antigens directly promote tumor progression by activating oncogenic signaling pathways (e.g., EGFR/FAK) and inducing epithelial-mesenchymal transition. Additionally, these antigens play a noticeable role in immune suppression in the tumor microenvironment. Tn antigens bind to macrophage galactose-specific lectin (MGL) on myeloid cells, while STn antigens interact with sialic acid-binding immunoglobulin-like lectins (Siglecs), collectively inhibiting natural killer cell cytotoxicity, dendritic cell maturation, and T cell activation. Changes in serum levels of glycoprotein tumor markers (e.g., CA15–3 and CA125) are associated with aberrant protein glycosylation in cancer cells, which may influence their expression levels, stability, or immunodetection. Current therapeutic approaches include monoclonal antibodies (e.g., Remab6, L2A5), antibody-drug conjugates, CAR-T cell therapies, and vaccines. However, challenges remain due to glycan heterogeneity and low immunogenicity.

**Conclusion:**

Tn/STn antigens play a pivotal role in tumorigenesis and immune evasion, presenting significant potential for both diagnostic and therapeutic applications. Future research should concentrate on elucidating the underlying mechanisms, developing innovative detection technologies, and promoting multidisciplinary collaborations to advance Tn/STn antigen-based tumor molecular subtyping, precision targeted therapies, and efficacy prediction systems, thereby providing new directions for cancer diagnosis and treatment.

## 1 Introduction

### 1.1 Aberrant O-glycosylation and tumorigenesis

**Figure 1:**
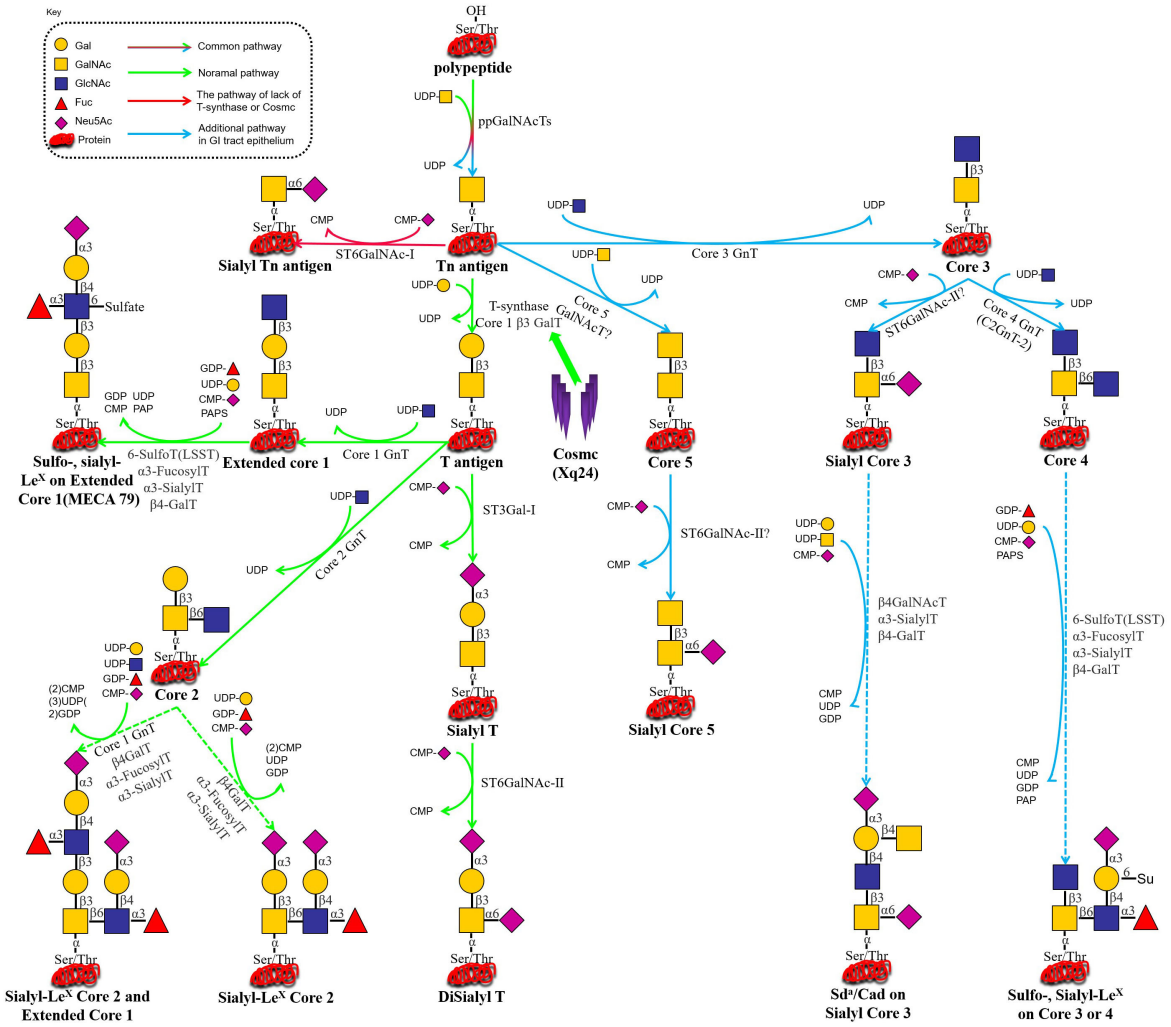
(i) *C1GALT1* (T-synthase) → T/core-1 → extension: Glycoprotein biosynthesis begins in the endoplasmic reticulum, where N-glycans are added via a co-translational process. While O-glycosylation typically initiates in the Golgi apparatus, where T-synthase adds galactose from UDP-Gal to the common precursor Tn antigen (GalNAcα1-O-Ser/Thr), yielding T antigen (Galβ1-3GalNAcα1-O-Ser/Thr). The T antigen can then be modified by multiple glycosyltransferases to form various types of extended structures. (ii) *C1GALT1*/COSMC loss → STn via *ST6GALNAC1* (ST6GalNAc-I): In the absence of functional COSMC (being essential for active T-synthase formation) or when functional T-synthase is lost through other unknown mechanisms, Tn antigen may react with ST6GalNAc-I, which transfers a Neu5Ac unit (from CMP-Neu5Ac) onto Tn antigen to form STn. Due to the low efficiency of ST6GalNAc-I, its overexpression is unlikely to inhibit T-synthase function and cause pathological STn expression (44, 45). (iii) *B3GNT6* (core-3) predominant in GI epithelium: In gastrointestinal epithelial cells, core-3 GnT transfers a GlcNAc unit (derived from UDP-GlcNAc) to the Tn antigen, forming a core-3 structure. This core-3 structure is further modified to form extended structures, including sulfo-LeX, sialo-LeX, or Sda on either the core-3 or core-4 backbones (46).

**Figure 2:**
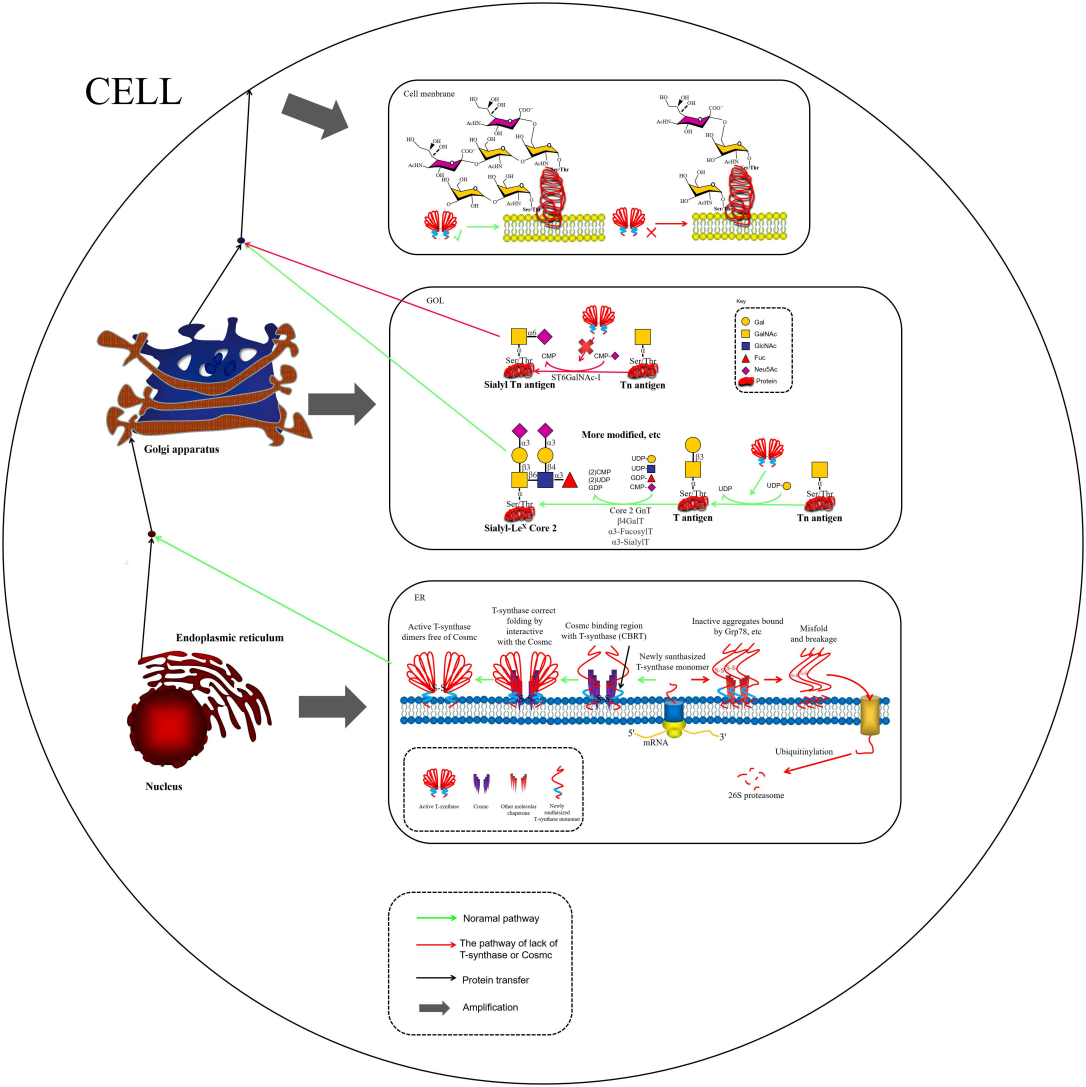
COSMC is an endoplasmic reticulum-localized molecular chaperone being critical for the proper folding of T-synthase. In the absence of COSMC, newly synthesized T antigen monomers may bind to other chaperones, such as Grp78, forming misfolded T antigens. These misfolded T antigens are subsequently ubiquitinated and degraded by the 26S proteasome (47). In the Golgi apparatus, active T-synthase utilizes Tn antigens to synthesize T antigens and additional extended structures. Tn antigens undergo ST6GalNAc-I modification to form STn Antigens. While STn antigens cannot undergo further glycosylation modifications(other glycosites can still be modified), their expression can lead to the overaccumulation of both Tn and STn antigens on the cell membrane.

Protein activity is heavily reliant on cellular signaling networks and post-translational modifications (PTMs). Protein glycosylation, a frequent type of PTMs, is directly regulated by the dynamic balance of the cellular metabolic state and sugar source molecules. Protein glycosylation is a highly regulated process that follows two primary pathways: N-glycosylation and O-glycosylation. N-glycosylation occurs at asparagine (Asn) residues in the consensus sequence Asn-X-Ser/Thr (where X ≠ Pro), and it begins in the endoplasmic reticulum (ER) and is completed in the Golgi apparatus. In contrast, O-glycosylation involves the attachment of glycans to serine (Ser) or threonine (Thr) residues, typically without a strict consensus sequence, and occurs predominantly in the Golgi apparatus, where monosaccharides are sequentially added to extend the glycan structure (**Figures 1** and **2**) (1). This covalent glycan attachment is not random, while is mediated by a series of glycosyltransferases, determining the precise structure of the glycan. These glycan modifications are essential for numerous biological processes, including protein folding, intracellular trafficking, intercellular interactions, immune surveillance, and regulation of cellular differentiation (2, 3). Importantly, changes in glycosylation dynamics directly influence the physicochemical properties and functional characteristics of proteins, acting as molecular switches that enable cells to respond to internal and external stimuli.

Aberrant O-glycosylation is a notable feature of malignant tumors during carcinogenesis and tumor progression. The altered glycan structures, known as tumor-associated carbohydrate antigens (TACAs), serve as critical biomarkers of cancer (4, 5). One such antigen is the Tn antigen (GalNAcα1-Ser/Thr), which is a truncated form of O-GalNAc-type mucinoglycans. The Tn antigen is the initial product of mucin-type O-glycosylation, catalyzed by polypeptide N-acetylgalactosaminyltransferases (GalNAc-Ts or ppGalNAc-Ts) (6). The synthesis of the Tn antigen is linked to a blockage in the normal O-glycosylation pathway. Under physiological conditions, the Tn antigen is extended by core 1 β1,3-galactosyltransferase (T synthase), forming the core 1 structure (Galβ1-3GalNAcα1-Ser/Thr, i.e., the T antigen), which is a critical intermediate for the subsequent formation of more complex O-glycan structures (7), as depicted in **Figure 1**. In cancerous cells, however, this pathway is disrupted, leading to the accumulation of Tn antigen. The Tn antigen can be further extended to the sialylated Tn antigen (Sialyl-Tn, STn) by the action of sialyltransferase ST6GalNAc-I. In the tumor microenvironment (TME), abnormal glycosyltransferase activity halts O-glycan synthesis at the Tn/STn stage, preventing the formation of fully branched O-glycans. While the Tn/STn antigens are minimally expressed in healthy tissues, they are significantly upregulated on the surface of malignant cells. Elevated expression of Tn/STn antigens is associated with increased tumor invasiveness, metastasis, and poor prognosis, highlighting their potential as both diagnostic markers and therapeutic targets in cancer (8–10).Furthermore, the activity of T synthase is entirely dependent on its molecular chaperone, COSMC, which is crucial for the correct folding of T synthase in the ER, thereby ensuring its catalytic function. In malignant tumors, O-glycosylation modifications mainly exhibit significant “synthetic truncation.” This is notable in approximately 80% of colorectal cancer cases, 57% of breast cancer cases, and 56% of pancreatic cancer cases, where the T synthase loses its function. This loss can result from genetic mutations (e.g., somatic mutations in COSMC), episodic silencing, or inhibition of enzyme activity, all of which impair the conversion of Tn antigen to T antigen. Consequently, the blockage of this glycosylation step leads to the accumulation of Tn antigen and contributes to the altered glycosylation profile associated with cancer progression (11–13). In TMEs where ST6GalNAc-I sialyltransferase is overexpressed, stationary Tn antigens are catalyzed to produce sialyl-Tn (STn) antigens, creating an irreversible sialylation termination signal (14), as illustrated in **Figures 1** and **2**.

Tumor-associated glycan antigens (TACAs) are distinctive sugar chain structures formed by abnormal protein glycosylation modifications in malignant tumors. They play a remarkable role in tumorigenesis and metastatic colonization by altering cellular signaling, mediating immune escape, and driving epithelial-mesenchymal transition (EMT) (4, 5, 10, 15–18). Mucin-type TACAs, particularly Tn and STn antigens, are characteristic of this altered glycosylation. In the mucin family (MUC1/2/4/6, etc.), O-glycan synthesis is halted at the Tn/STn stage due to an imbalance in the glycosylation enzyme network during cancerous transformation. This results in the accumulation of truncated, exposed glycan chains, which are pathologically altered and recognized by lectin receptors (e.g., Siglec and Galectin). These interactions initiate the “TACA-glycose-binding receptor (GBR) axis,” promoting an immunosuppressive microenvironment and enabling tumor cells to evade immune surveillance by T/NK cells (19–25). The Tn antigen has shown to promote proliferative invasion in colorectal cancer cells (26, 27) and pancreatic cancer cells (28) by activating the EGFR/FAK signaling pathway. Additionally, the Tn antigen is prevalent in breast cancer and correlates with lymph node metastasis and poorer survival outcomes (29). The STn antigen contributes to immune evasion by masking immunorecognition epitopes and is found in approximately 80% of human malignancies, where it promotes cancer progression and aggressiveness (12, 30, 31). A recent study found that Tn and STn antigens are highly expressed in esophageal adenocarcinoma (EAC) (about 71% for both), establishing them as tumor-specific markers for EAC (32). Furthermore, the presence of Tn/STn antigens in various solid tumors, including ovarian cancer, prostate cancer, and lung cancer, has been linked to poor clinical outcomes, highlighting their potential as diagnostic and therapeutic targets in cancer (33–35).

### 1.2 New paradigms in glycosylation research: engineering design and artificial intelligence-driven strategies

**Figure 3:**
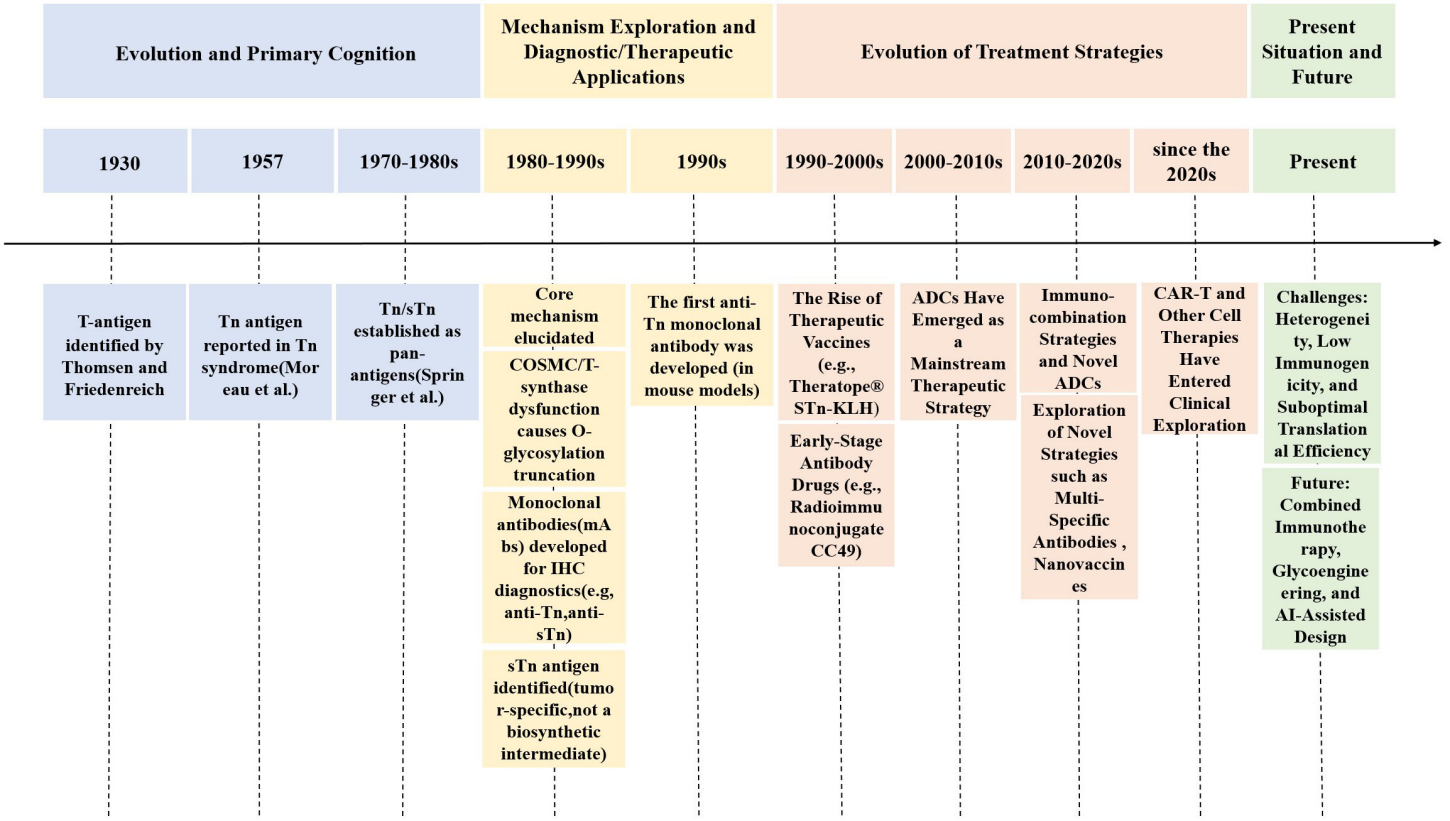
This diagram illustrates the century-long evolution of research on tumor-associated glycan antigens, particularly Tn/STn antigens. It involves the identification of the T antigen in 1930 and the first identification of the Tn antigen in 1957, in order to the elucidation of underlying mechanisms, such as abnormal O-glycosylation caused by the inactivation of the molecular chaperone COSMC. The timeline progresses through significant milestones, including the use of monoclonal antibodies in immunohistochemical diagnostics, the development of therapeutic vaccines (e.g., Theratope^®^), and the rise of antibody-drug conjugates (ADCs) as mainstream therapeutic strategies. Further advancements have led to contemporary explorations, including immune combination therapies, multispecific antibodies, and CAR-T cell therapies. The field faces challenges, such as tumor heterogeneity and low immunogenicity, while future directions will concentrate on innovations in glycoediting and AI-assisted design.

In recent years, glycan research has undergone a paradigm shift from traditional characterization toward engineered design and AI-driven discovery (**Figure 3**). Regarding glycoengineering platforms, a core advancement involves precisely programming glycan chains using synthetic biology. For instance, GlycoEra’s CustomGlycan platform employs the genetic modification of cell factories, such as Chinese Hamster Ovary (CHO) cells, through the knockout of native glycosyltransferases and the introduction of custom-designed toolkits. This methodology promotes the production of specific glycan chains with a homogeneity of 99.7%. Additionally, the platform’s dual-functional degraders are capable of eliminating more than 95% of pathogenic antibodies within 30 min (36). Recent research has reported the development of an innovative, highly efficient, and stereoselective α-glycosylation method, enabling the systematic and efficient synthesis of a molecular library comprising 13 key TACAs and O-glycans from a single intermediate (37). This approach provides a robust supply of structurally characterized, pure glycan molecules, which hold significant potential for applications in cancer vaccine development, glycoimmunology, and the creation of diagnostic tools. The scientific advancements demonstrated by these methodologies highlight their remarkable value and promise. Furthermore, the integration of AI has substantially accelerated the analysis of previously uncharacterized glycan structures. Yan Ning’s team developed the “CryoSeek” strategy, integrating cryo-electron microscopy (cryo-EM) with AI-assisted automated modeling. This approach enables the direct identification and resolution of atomic structures of previously uncharacterized glycoprotein fibers from natural environmental samples, thereby highlighting the crucial role of glycans in maintaining biological assembly structures (38, 39), Additionally, software, such as PEAKS GlycanFinder employs deep learning-based algorithms to effectively address the challenge of fuzzy matching for glycosylation sites and glycan structures, thereby significantly improving identification sensitivity (40). Advanced analytical techniques, including glyco-reductive end dual isotope labeling (GREDIL), deep quantitative glycoprotein analysis (DQGlyco), automated glycosylation optimization, and enhanced mass spectrometry strategies, have significantly enhanced our ability to deeply decipher glycosylation heterogeneity and dynamic alterations (41–43). The convergence of these approaches is driving the field of sugar science toward greater precision and predictability.

## 2 Tumor-associated Tn and STn antigen production routes and upregulation mechanisms

### 2.1 Tn/STn antigens: formation, regulation, and pathological implication

The molecular mechanism of protein glycosylation modification is based on a highly organized enzymatic cascade reaction, with the key steps regulated by a family of Golgi-localized glycosyltransferases. In the mucin-type O-glycosylation process, the synthesis of the Tn antigen marks the initiation of glycan chain formation. Polypeptide N-acetylgalactosaminyltransferase (pp-GalNAc-T) catalyzes the attachment of N-acetylgalactosamine (GalNAc) from the UDP-GalNAc donor to the polypeptide chain via α-glycosidic bonds. This reaction is directed by specific serine/threonine sequence motifs, yielding the Tn antigen (GalNAcα1-Ser/Thr), representing the first step in O-glycosylation modification (48). Subsequently, T synthase in the Golgi apparatus forms the T antigen by linking galactose (Gal) to GalNAc through β1–3 glycosidic bonds, resulting in the core 1 disaccharide structure (Galβ1-3GalNAcα1-Ser/Thr). The enzymatic activity of T synthase is critically dependent on the quality regulation of its protein folding by the molecular chaperone COSMC in the ER (49, 50), as depicted in **Figure 1**. COSMC prevents T synthase from misfolding and aggregating in the ER (47, 51, 52). In the absence of COSMC, the ER retention protein GRP78 recognizes T synthase and mediates its re-trotranslocation to the cytoplasm, where it is targeted for degradation via the ubiquitin-proteasome pathway (**Figure 1**) (35, 47, 53, 54). Additionally, the sialic acid transfer enzyme ST6GalNAc-I catalyzes the transfer of α2,6-sialic acid (Neu5Ac) to GalNAc residues on the Tn antigen, generating the sialyl-Tn (STn) antigen (Neu5Acα2-6GalNAcα1-O-Ser/Thr), a modification that irreversibly terminates glycan chain extension (55). These two competing pathways, T antigen synthesis and sialylation, govern the metabolic function of Tn antigen during physiological O-glycosylation. When T synthase activity is compromised, such as through COSMC mutations, or when ST6GalNAc-I is overexpressed, glycan synthesis is biased toward the STn pathway, leading to the abnormal accumulation of Tn/STn antigens. Moreover, β1,3-N-acetylglucosaminyltransferase (*B3GNT6*) and β1,6-N-acetylglucosaminyltransferase (*GCNT3*) can extend the Tn antigen to form Core 3 (GlcNAcβ1-3GalNAcα1-) and Core 4 (GlcNAcβ1-6 (GlcNAcβ1-3) GalNAcα1-) structures. Notably, Core 3 glycans are essential for maintaining the integrity of the intestinal epithelial mucus barrier and preventing the onset of colorectal inflammatory carcinogenesis (**Figure 1**) (56, 57). Pathological accumulation of Tn/STn antigens in malignant tumors involves multilevel regulatory disruptions, as summarized in **Table 1**.

**Table 1:** Classes of pathologic accumulation mechanisms for Tn/STn antigens.

Type of mechanism	Molecular biology	Representative disease associations
Synthetic pathway blocking	• COSMC somatic mutation and hypermethylation → T synthase misfolding (58) • COSMC/T synthase episodic silencing (59, 60)	Pancreatic cancer Tn syndrome
Competitive suppression	•ST6GalNAc-I overexpression → preferentially catalyzes STn generation (61)	Breast cancer Ovarian cancer
Dysregulation of enzyme activity	• Abnormally high ppGalNAc-T expression/localization shift → uncontrolled Tn production (62)	Stomach cancer Prostate cancer
Decreased substrate availability	• Defective UDP-GalNAc transport → scarcity of substrates for glycan chain extension (63)	Hepatocellular carcinoma
Microenvironmental perturbation	• Lysosomal acidification (pH 5.0-6.0) → inhibition of glycosyltransferase activity (6)	Solid tumor microenvironment

The foregoing methods exhibit dynamic synergy in several tumor types; for example, in colorectal cancer, COSMC mutations and salivary transferase ST6GalNAc-I overexpression frequently coexist, resulting in STn accumulation via a “synthetic blockade + competitive inhibition” (64). This multifactorial pattern of interactions demonstrates that targeted therapy against Tn/STn antigens should be designed using molecular typing of specific cancer types to develop a combined intervention strategy. In lung cancer, high ST6GalNAc-I expression level drives STn glycosylation of MUC5AC. Acting as a “molecular glue,” STn-MUC5AC aberrantly binds to NECTIN2, enhancing tumor cell collective migration and survival, thereby promoting invasion and metastasis (65). This study presented valuable mechanistic insights into the biological role of the STn antigen and provided guidance for the development of novel diagnostic and therapeutic strategies targeting cancer metastasis. It should also be noted that the sole overexpression of *ST6GALNAC1* rarely overrides the function of an active T-synthase. Due to its relatively low catalytic efficiency, even under overexpressed conditions, the normal O-glycosylation pathway takes precedence as long as the T-synthase pathway remains intact and active. Consequently, this hierarchical preference effectively restricts the abnormal accumulation of the STn antigen (44, 45, 66).

### 2.2 Pathological effects of molecular chaperone Cosmc dysfunction

The activity of T-synthase is strictly dependent on its molecular chaperone Cosmc, which assists in the proper folding of T-synthase within the endoplasmic reticulum. This step is essential for the subsequent synthesis of complex O-glycan branches (47). Loss of function in the COSMC-T-synthase axis significantly enhances tumor progression by altering the protein glycosylation profile, arresting O-glycan synthesis at the Tn antigen stage, and triggering multiple pathological effects. Gut-specific Core 3 O-glycan synthesis impairment leads to loss of mucus layer integrity, promoting inflammation-associated carcinogenesis (56, 57). In a pancreatic ductal adenocarcinoma (PDAC) model, it has been demonstrated that approximately 40% of cases exhibited COSMC gene promoter hypermethylation, leading to the silencing of COSMC expression. This, in turn, results in the downregulation of T-synthase and overexpression of Tn/STn antigens. The resulting glycosylation imbalance disrupts E-cadherin-mediated cell adhesion, promotes EMT, and enhances tumor cell invasiveness and metastatic potential (6). Abnormal COSMC function, as the only known regulator of T-synthase activity, is closely associated with malignant tumors, such as pancreatic cancer and colorectal cancer, as well as non-neoplastic diseases, including IgA nephropathy and Tn syndrome. The core pathological mechanism underlying these conditions is protein glycosylation truncation, leading to cell surface glycan remodeling and the reprogramming of microenvironmental signaling pathways. This mechanism provides a theoretical basis for the development of targeted therapies based on glycan editing. Furthermore, a study demonstrated that upregulation of T-synthase significantly inhibited osteosarcoma cell proliferation *in vitro*. Osteosarcoma cells with elevated T-synthase expression also promoted the proliferation of CD8+ T cells, inhibited apoptosis, and induced a remarkable elevation in cytotoxic T lymphocyte (CTL) growth through elevated IFN-γ production (67). This outcome provides new directions for clinical immunotherapy strategies in osteosarcoma.

## 3 Mechanisms of action of Tn and STn antigens in association with the tumor immune microenvironment

### 3.1 Mechanisms of Tn antigen and immune microenvironment interactions

**Figure 4:**
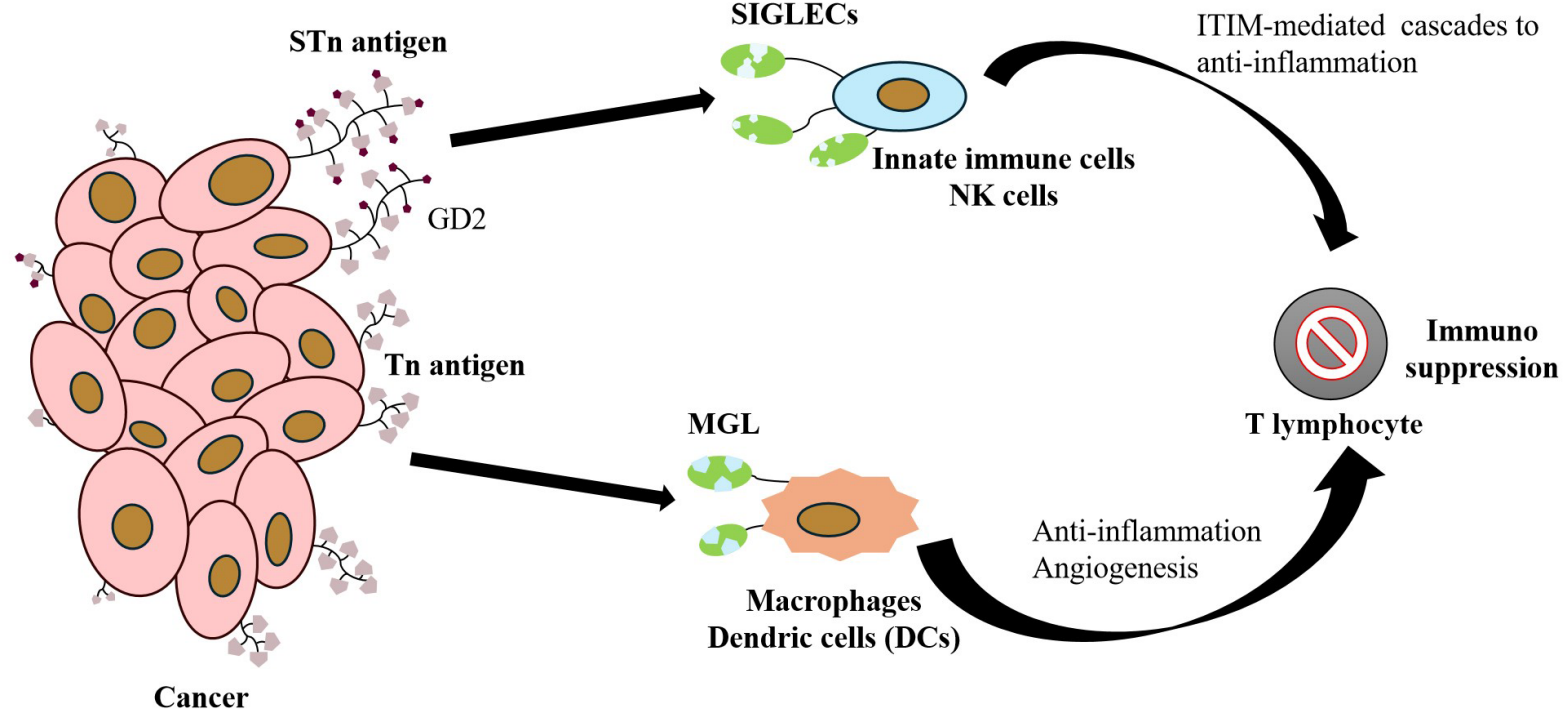
Core Mechanism of Tumor Cell Immune Evasion Mediated by Surface-Specific Glycoantigens: The Tn antigen highly expressed on cancer cells binds to the MGL receptor on the surface of macrophages/dendritic cells, inducing an anti-inflammatory phenotype and promoting angiogenesis. Concurrently, GD2 and STn antigens interact with SIGLEC family receptors on innate immune cells, such as NK cells, activating inhibitory cascades via ITIM signaling domains. This interaction synergistically suppresses immune cell function, thereby promoting the establishment of an immunosuppressive microenvironment that accelerates tumor growth.

Notably, TME is a dynamic ecosystem composed of tumor cells, stromal cells, and infiltrating immune cells, and the complex molecular interactions among its components dominate malignant biological behaviors, such as tumorigenesis, metastasis, and treatment resistance. As the core functional unit of TME, tumor immune microenvironment (TIME) contains immune cell subpopulations, including macrophages, natural killer (NK) cells, dendritic cells (DCs), and T/B lymphocytes, which regulate the balance of tumor immune surveillance and escape through immune checkpoint signaling and cytokine secretion (**Figure 4**) (68). At the molecular level, the intercellular communication in the TME is heavily influenced by protein glycosylation modifications. Glycoconjugates, including glycoproteins, proteoglycans, and glycolipids, are found on nearly all cell surfaces and play a remarkable role in maintaining microenvironmental homeostasis. These glycosylation structures mediate cellular recognition, signal transduction, and immune-antigen presentation (69–71). Tn antigen, a prototypical marker of abnormal O-glycosylation, promotes tumor progression through a dual mechanism. Firstly, it activates the EGFR/FAK signaling pathway, leading to increased tumor proliferation, apoptosis inhibition, and enhanced migratory invasiveness (6, 26, 28). Secondly, Tn antigen binds to macrophage galactose-type lectin (MGL/CD301), a receptor predominantly expressed on myeloid immune cells, particularly tolerogenic DCs and M2-type macrophages. MGL shows high specificity for recognizing tumor-associated Tn/STn antigens, and its binding subsequently impairs NK cell cytotoxicity, blocks DC maturation, and induces regulatory T cell (Treg) expansion (**Figure 4**) (72–76). This immunoediting mechanism, driven by glycan-lectin interactions, highlights the role of MGL as a biomarker for distinguishing tumor-specific glycan epitopes from those in normal tissues. Additionally, myeloid cell surface pattern recognition receptors, by specifically recognizing truncated glycan chains, contribute to immune evasion. MGL’s cross-recognition of low-protein glycosylated Tn/STn antigens and microbe-associated glycan chains constitutes a molecular link between tumor progression and the microbiome (74). This interaction inhibits M1 polarization of TAMs, blocks maturation of mononuclear DCs (moDCs), and reduces NK cell activity, providing a myeloid-dominated immunosuppressive microenvironment. The mannose receptor (CD206), which is highly expressed on TAMs in ovarian cancer, binds to the MUC16-STn complex, resulting in the increased secretion of the anti-inflammatory cytokine IL-10 and suppression of T-cell chemokine CCL3. This interaction significantly alleviates the anti-tumor immune response (77, 78). Mucins, including MUC1 and MUC16, are overexpressed in various gastrointestinal cancer types, and one of the key mechanisms by which they promote tumor survival is through immune evasion, effectively providing “protection” from immune cell-mediated killing (79–82). Specifically, MUC1-Tn interacts with MGL on the surface of antigen-presenting cells (APCs), leading to enhanced PD-L1 expression and activation of the T-cell depletion pathway. In colon cancer, MUC1-Tn has shown to promote regulatory T cell (Treg) expansion and inhibit CD8+ T cell proliferation through APC-MGL interactions. Furthermore, Tn antigens on MUC1 are recognized by MGLs on macrophages and DCs, playing a critical role in tumor progression (76, 83). Given these mechanisms, liquid biopsy markers, such as CA15-3 (MUC1) and CA125 (MUC16) have demonstrated strong diagnostic potential in breast cancer and ovarian cancer (84–86). Additionally, a synergistic anti-tumor effect has been found with the combination of an anti-MUC1-Tn monoclonal antibody (mAb) and a PD-1 inhibitor in colon cancer (76).

Recent studies have demonstrated that tumor cell surface Tn antigens are specifically recognized by MGL receptors on APCs, establishing a key mechanism for immune evasion across various cancer types. In colorectal cancer cells, Tn antigen overexpression induced by shRNA-mediated COSMC gene silencing significantly inhibited CD8+ T-cell tumor infiltration and promoted the expansion of FoxP3+ Treg cells, forming an immunologically “cold” microenvironment (87, 88). In a Tn-overexpressing lung adenocarcinoma model, tumor cells interacted with MGL+ APCs, triggering the secretion of the tolerogenic cytokine IL-10 and inhibition of IFN-γ production, leading to activation of the PD-1/PD-L1 axis (89, 90). Studies on PDAC have reported that dual activation of MGL (Tn antigen recognition) and DC-SIGN (fucoidan glycosylation antigen recognition) on the surface of TAMs drives an imbalance in the IL-10/IL-6 ratio and establishes a resistant microenvironmental signature. However, it has been reported that a monoclonal antibody targeting the galactoglucan-3 binding protein (Gal-3BP) in a PDAC-like organ model eliminated the transfer of PDAC cells *in vivo* (19, 91, 92). A recent study on PDAC has indicated that nanomaterial-assisted delivery of immune checkpoint inhibitors can enhance antitumor immunity while minimizing side effects, highlighting their potential as a novel immunotherapeutic strategy (93).

Although the potential role of the Tn antigen in tumor immunoediting has gained initial attention, whether it serves as a central mechanism to mediate immune escape from cancer cells *in vivo* remains controversial. Multiple studies were conducted to reveal the dual regulatory effects of Tn antigen accumulation on tumor biological behavior through gene editing models, and CRISPR/Cas9-mediated knockdown of the *C1GALT1C1* gene has been reported to alter the cell-intrinsic properties of tumor cells, influencing genes related to MAPK signaling, cell migration, angiogenesis, and immunomodulation (87). Previous reports have demonstrated that knockdown of T synthase significantly increased galectin-1 secretion both *in vivo* and *in vitro*, and notably enhanced the production of Th2 cytokines (IL-10 and IL-4) *in vivo*. Furthermore, the T synthase knockdown-induced rise in galectin-1 level promoted apoptosis of CD8+ T cells (19, 94). This immunosuppressive mechanism, which supports tumor cell persistence, migration, and invasion, presents potential diagnostic and therapeutic targets for cancer treatment.

### 3.2 Mechanisms of STn antigen and immune microenvironment interactions

The STn antigen (Neu5Acα2-6GalNAcα1-O-Ser/Thr) is synthesized via a specific enzymatic reaction mediated by ST6GalNAc-I. Sialyltransferase catalyzes the α-2,6-sialylation of the GalNAc residue on the Tn antigen, generating a terminal glycoepitope with immunoregulatory properties (55). Previous research using colon cancer models has confirmed that mucin-type STn antigens obscure the binding sites of NKG2D ligands (e.g., MICA/B) via spatial site-blocking effects, resulting in a significant reduction in the release of cytotoxic particles, thereby establishing a molecular mechanism of immune evasion (95). The STn antigen serves as a high-affinity ligand for salivary acid-binding immunoglobulin-like lectins (Siglecs). Among the 14 human Siglec isoforms, STn preferentially binds to those containing the ITIM structural domain (e.g., Siglec-7, -9, -10, -15), triggering a SHP-1/SHIP-1 phosphatase pathway that inhibits TCR/CD28 co-stimulatory signaling, thereby mediating immunosuppressive mechanisms (68, 96), as illustrated in **Figure 4**. Pathological accumulation of STn antigen in gastrointestinal tumors exhibited multilayered regulatory features, interfering with the normal O-glycosylation process through competitive inhibition of T synthase activity and activation of the ST6GalNAc-I autocrine loop, resulting in the overexpression of the enzyme and driving the pathological phenotype of hypersialylation at the terminal end of the sugar chain (14).

Notably, PDAC tumor cell surface STn antigen interacts with Siglec-7 and Siglec-9 on monocytes/macrophages, driving phenotypic transformation of TAMs and inducing the secretion of pro-angiogenic factors. These factors, in turn, promote angiogenesis and decrease the infiltration density of CD8+ T cells, accelerating tumor malignant progression (97). In colon cancer, tumor-infiltrating mast cells express Siglec-6, identified as a hypoxia-responsive immunosuppressive receptor (HIF-1α-dependent). Binding of Siglec-6 to sialylated ligands on the tumor cell surface leads to a significant reduction in the efficiency of cytotoxic granule release from mast cells, a pathological process that is dose-dependently enhanced in the hypoxic microenvironment typical of solid tumors (98). Siglec-9, carrying ITIM/ITIM-like structural domains, exhibits differential regulation in myeloid-lymphoid lineage cells. In monocytes and NK cells, the MUC16-STn antigen-Siglec-9 ternary complex activates the immune checkpoint network, inhibiting NK cell activation and correlating with reduced ovarian cancer patient survival. In the MUC1+ TME, Siglec-9 on myeloid cells binds to α2,3-sialylated glycoepitopes, promoting the conversion of TAMs to a pro-angiogenic phenotype via the Syk-PI3K/AKT signaling pathway, thereby establishing a dual metabolic-immune suppressive niche (99). In the study of hepatocellular carcinoma (HCC) microenvironment, a subpopulation of Siglec-10-high TAMs in HCC tissues was significantly correlated with patients’ shortened overall survival, as revealed by single-cell transcriptome sequencing (scRNA-seq). These TAMs create a T-cell depletion ecosystem through the upregulation of immune checkpoint molecules, such as PD-1, CTLA-4, and TIM-3. Targeting Siglec-10 with monoclonal antibodies effectively blocks this negative regulatory network, leading to a remarkable elevation in IFN-γ and IL-2 secretion, and synergistically enhancing CD8+ T cell anti-tumor cytotoxicity (100). In the PDAC microenvironment, Siglec-15 expressed by TAMs specifically binds to tumor cell α2,6-sialylated STn antigens and α2,3-sialic acid-modified glycans. These interactions induce M2-type polarization markers through activation of the Syk-PI3K/STAT6 signaling pathway. Notably, sialidase-mediated removal of glycan end modifications significantly decreases TAM reprogramming efficiency, impairing their tumor-promoting function (101, 102).

**Figure 5:**
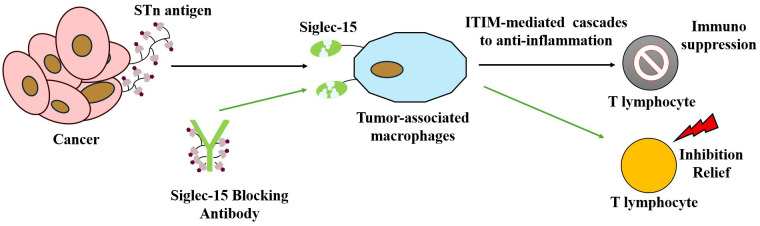
Illustrating how tumor cells express salivary glycan ligands that bind to Siglec-15 on macrophage surfaces, thereby suppressing T cell activation and function to achieve immune escape. This figure also highlights a targeted therapeutic strategy: anti-Siglec-15 blocking antibodies bind Siglec-15, thereby reversing its inhibitory effect on T cells and restoring T cell-mediated antitumor immune responses.

The STn-Siglec interaction network functions as a critical glyco-immune checkpoint that enhances tumor immune escape, thereby promoting tumor progression. Additionally, salivary glycan ligands can inhibit effective T-lymphocyte activation by binding to Siglec-15 (103). This interaction induces hypersialylation and promotes immunosuppression, contributing to immune evasion by tumor cells (104), as depicted in **Figure 5**. Thus, Siglec-15 remains a promising immune checkpoint protein, with differential expression observed in various cancer subpopulations, providing potential new therapeutic targets for cancer treatment.

## 4 Precision diagnosis and treatment of cancer patients based on Tn and STn antigens

### 4.1 Advances in targeting Tn/STn antigens for diagnosis and therapy

Aberrant O-glycosylation is a prominent molecular feature of malignant tumors. Through the formation of structurally abnormal O-glycan fragments, including the Tn antigen and its sialylated derivative, the STn antigen, is frequently noteworthy, with significantly elevated expression level found in over 80% of epithelial tumors. These glycoepitopes are not only associated with poor prognosis and metastasis, but also serve as potential biomarkers for tumor progression (10). Previous research has demonstrated a correlation between Tn and STn antigens and various tumor types (105). Numerous clinical studies have pointed out that these truncated glycoepitopes function as independent predictors of tumor invasiveness, metastatic potential, and poor prognosis. Additionally, they are directly implicated in key processes of tumorigenesis and progression (5, 15, 18). The tumor-promoting effects of Tn and STn antigens are driven not only by the malignant transformation of cells due to aberrant glycosylation, but also by their immune regulatory functions. The specific binding of tumor-associated antigens to the MGL receptor on macrophages and DCs can remodel the TME, inducing an immune-tolerant phenotype and promoting the establishment of a malignant TME both within and around the tumor tissue, as illustrated in **Figure 4** (20, 106). Over the past decade, the association between STn/Tn antigens and tumors has been progressively elucidated, with the core molecular mechanism involving the inactivation of COSMC/T-synthase leading to abnormal O-glycosylation (107). Recent studies have validated the feasibility of targeting these antigens with antibodies and novel vaccines using *in vivo* models, such as tumor-bearing mice to demonstrate therapeutic potential.

In the late 20th century, Professor Georg Springer’s research was the first to utilize antiserum against the natural anti-T antigen for detection, revealing that the Tn antigen was expressed in the majority of breast cancer tissues, while was rarely found in normal breast tissue (108). The STn antigen has also been identified as abnormally overexpressed in breast cancer, correlating with lymph node metastasis, high histological grade, and chemotherapy resistance (109). The Theratope vaccine, developed based on these findings, failed to significantly extend progression-free survival (PFS) or overall survival in a Phase III clinical trial for metastatic breast cancer (110). This lack of efficacy may stem from its inability to induce a robust cytotoxic T lymphocyte response, its susceptibility to endogenous glycosidases, and its tendency to elevate IgM level in patients without eliciting a strong IgG response or long-term immune memory. The failure of Theratope highlights that stimulating an immune response at the biomarker level does not necessarily translate into clinical benefit. Tumor heterogeneity, the immunosuppressive TME, and the nature and quality of the immune response induced by vaccines all present significant challenges to the success of cancer vaccines. This trial has provided valuable insights for future cancer vaccine research. Recent studies have proposed using vancomycin-binding lectin (VVL)-labeled nanoparticles for the fluorescent detection of the Tn antigen in breast and prostate cancer cells, presenting a novel diagnostic tool for these cancer types (111). Although research on STn/Tn-targeted therapies for prostate cancer remains limited, immunotherapy strategies targeting these antigens represent a promising area for exploration. However, the clinical translation of such approaches faces similar challenges to those encountered in breast cancer.

During the 1980s and 1990s, the association between the STn/Tn antigens and various cancer types, including lung cancer, gradually gained clinicians’ attention. Using techniques, such as immunohistochemistry, researchers identified abnormal expression of the Tn antigen across multiple cancer types, including lung adenocarcinoma. This expression was found to be closely associated with tumor progression, increased invasiveness, and poorer patient prognosis (112). Based on Shuvalova et al.’s research, COSMC-knockout A549 lung adenocarcinoma cells expressed Tn-antigen-modified CD44 that was specifically detected by the AKC3 antibody. These results indicate that Tn-CD44 may serve as a novel biomarker for non-MUC1-expressing lung cancers and provide a proof-of-concept rationale for developing AKC3-based diagnostics and CAR-T therapies (113, 114). More recently, Huo et al. reported the development and evaluation of a novel cancer vaccine based on N (OMe)-linked sialic acid-Tn (STn) antigen, which demonstrated a significant enhancement of antitumor immunity (115). This approach holds noticeable potential for advancing other TACA-based cancer vaccines, providing valuable insights and experimental evidence for the design of next-generation glycosylated cancer vaccines.

### 4.2 Breakthroughs in multimodal antibody development targeting Tn/STn antigens: from preclinical research to clinical translation

In the field of glycoproteomics biomarker development, substantial clinical breakthroughs have been achieved in targeting strategies for protein glycosylation-dependent epitopes. Clinically validated tumor antigens or glycoprotein biomarkers identified using specific detection methods include CA19-9, CA15-3, and CA125, relying on MUC16 core O-GalNAc modification, as well as CA72-4, specifically recognizing Tn antigen clusters (116). However, the central bottleneck in this field arises from the immunogenicity gradient differentiation of antigenic epitopes induced by protein glycosylation heterogeneity. This phenomenon leads to systematic epitope preferences during antibody screening. Approximately 30 monoclonal antibodies targeting Tn/STn antigens are currently in the development pipeline, several of which have demonstrated significant antitumor activity in preclinical models (117). Representative examples include the IgG1 83D4 antibody, which specifically binds the GalNAcα1-O-Ser/Thr conformational epitope, exhibiting subcellular localization specificity (118); the MLS128 antibody, which selectively targets the Tn-MUC1 glycopeptide complex, promoting tumor tissue-specific binding (119); and the 5E5 antibody, which primarily targets the Tn antigen while exhibiting minimal cross-reactivity with the STn antigen (120, 121).

The research and development of monoclonal antibodies targeting Tn/STn antigenic epitopes has formed a multimodal therapeutic technology system, and its application scenarios cover multiple dimensions, such as tumor screening, targeted therapy, and immune modulation. For instance, the monoclonal antibody TKH2, which specifically recognizes the STn antigen epitope, functions as the targeting component in a cisplatin-loaded polymeric nanodelivery system. This system significantly enhances the chemotherapeutic sensitization effect of gemcitabine in STn-highly expressed PDAC cells (122). In the field of radioimmunotherapy, the second-generation humanized IgG1κ anti-TAG-72 antibody CC49, after being labeled with lutetium-177, has entered the clinical trial phase for ovarian cancer treatment in combination with interferon-α and paclitaxel (123). Notably, innovative therapies targeting immune checkpoints are progressing rapidly. The NC318 monoclonal antibody, in Phase I/II clinical trials (NCT03665285), provides a novel treatment strategy for metastatic solid tumors, such as colorectal cancer and cholangiocarcinoma, by blocking the Siglec-15 signaling pathway (124). Furthermore, the Gatipotuzumab monoclonal antibody (NCT03360734/NCT01222624) has demonstrated the ability to activate antibody-dependent cell-mediated cytotoxicity (ADCC) by inhibiting the interaction between STn-MUC1 and Siglec-9 (125).

Previous research demonstrated an adjuvant-free, polylactic acid-hydroxyacetic acid copolymer (PLGA)-based nanoglycoconjugate antigen delivery system, which was designed to formulate a nanocandidate vaccine that induces the production of IgG specifically targeting MUC16 and MUC16-Tn glycoproteins, particularly concentrating on cancer cells and tumors. This approach presents remarkable promise for precise cancer targeting (126). Furthermore, a recombinant chimeric anti-Tn human IgG1 monoclonal antibody, Remab6, in conjunction with the mouse-derived IgM antibody ReBaGs6, has demonstrated efficacy in biochemical characterization of cancer cells and tumor immunohistochemical analysis. Notably, Remab6 exhibits a distinctive binding characteristic, in which its recognition of the Tn biomarker is independent of the IgA1 recognition pathway (123). This monoclonal antibody showed exceptional specificity for malignant tumor tissues, such as those found in gastrointestinal cancer and breast cancer, while minimizing non-targeted binding to normal tissues (120, 127). The glycoengineered variant, Remab6-AF (afucosylated), has shown to elicit potent ADCC against Tn-positive colorectal and breast cancer cell lines in preclinical studies (128). Moreover, Remab6 targets a diverse array of Tn+ glycoproteins, including non-mucin substrates, and recognizes partial epitopes that overlap with the MGL receptor binding domain (129). This multifaceted recognition property represents a novel approach to overcome TME-mediated immunotherapeutic resistance (130).

Additionally, recent advancements in the development of a novel preclinical anti-STn monoclonal antibody (mAb), L2A5 fragment antigen (Fab), have demonstrated its specific binding affinity for the core STn fragment (131). This enhancement of the anti-STn antibody provides promising potential for both diagnostic and therapeutic applications in tumors that exhibit high expression levels of STn antigens. A recent breakthrough has led to the development and validation of the L2A5 monoclonal antibody, targeting both the STn antigenic epitope and the α2–6 sialylated core 1 structure. L2A5 exhibits unique dual epitope recognition and demonstrates superior detection sensitivity, displaying a gradient of reactivity in tissue specimens of colorectal cancer across various stages of progression. Importantly, experimental data confirm that the binding affinity of L2A5 surpasses that of conventional anti-STn antibodies (132). Further analyses revealed that L2A5 could maintain high reactivity from the hypodifferentiated regions of tumors to the invasive front, highlighting its potential utility in analyzing tumor heterogeneity and advancing integrated diagnostic-therapeutic strategies.

Therapeutic strategies targeting tumor-associated carbohydrate antigens, such as Tn/STn, are making notable advancements across a variety of innovative approaches. In the field of cell therapy, Minerva Therapeutics’ huMNC2-CAR44 (NCT04025216) has demonstrated promising preliminary efficacy in early-phase clinical trials targeting solid tumors, with partial tumor responses found in a subset of patients (133). Meanwhile, the development of antibody-drug conjugates (ADCs) has flourished. By 2024, 13–15 ADC drugs had been approved globally, resulting in a remarkable accumulation of clinical experience and regulatory insights. This progress has increasingly clarified the regulatory pathways for therapeutic agents targeting glycan antigens, including ADCs, vaccines, and cell-based therapies. Although the development of highly specific anti-Tn/STn antibodies continues to face challenges related to immunogenicity, emerging technological platforms are making remarkable strides. For instance, novel phage display libraries employing a VH-dominant/VL-diversified strategy have enabled the isolation of antibodies capable of distinguishing closely related glycan isomers, such as Tn versus STn antigens, with evidence suggesting that the VL domain plays a key role in this specificity. In select cases, these antibodies also exhibit context-dependent recognition, discriminating between glycoprotein carriers bearing the same glycan (134). While this represents a significant advance toward addressing the long-standing challenge of generating glycan-isomer-specific antibodies, further validation is required to assess the generality and therapeutic potential of such context selectivity. Antibodies, such as AM52.1, developed by the Abrantes team, exhibit high specificity for the STn antigen without cross-reactivity to normal tissues, thereby providing a promising direction for solid tumor CAR-T therapy that combines both target specificity and safety (135). Collectively, these advancements are accelerating the translation of glycan antigen-targeted therapies from preclinical research to clinical application.

## 5 Summary and outlook

Although the correlation between aberrant expression of Tn/STn antigens and tumor progression has been widely demonstrated and the technical barriers to generating specific monoclonal antibodies have been largely overcome, the spatiotemporal specificity of its regulatory network remains to be elucidated, and Tn/STn-based liquid biopsy techniques are still challenged by glycoepitope heterogeneity and low abundance. The α-linked GalNAc, which forms the core structure of the Tn antigen, is relatively immunologically inert and may not be efficiently processed by the MHC-II T cell-dependent immune response, presenting a noticeable challenge to the development of effective antibodies (130). Another complication arises from the potential cross-reactivity of anti-Tn monoclonal antibodies with α-linked GalNAc-terminal structures, such as those found in blood group A (BgA) and Forssman antigens (136, 137), which may lead to false-positive serodiagnostic results. Additionally, circulating human immunoglobulin A1 (IgA1) contains Tn antigens within its hinge region (138–140), further complicating the specificity of diagnostic assays. Nonetheless, the development of specific anti-Tn monoclonal antibodies continues to be actively pursued as a valuable tool for cancer diagnosis and therapy. Research into tumor-associated glycan antigens (TACAs) is undergoing a critical transition from basic science to clinical application. A recent study introduced two novel probes based on uridine-5’-diphosphate-α-d-galactose (UDP-Gal) derivatives, along with a new enzyme labeling strategy, enabling the visualization, enrichment, and site-specific localization of Tn antigens with exceptional sensitivity and specificity (141). Additionally, a new method known as MOTAI provides a robust tool for in-depth analysis of O-GalNAcylation and complex O-glycosylation (142). The application of these advanced tools may significantly enhance our understanding of the biological functions of TACAs and promote the development of more accurate cancer diagnostic and therapeutic strategies. It is noteworthy that through the integration of mechanistic insights, technological innovations, and clinical advancements, a new standard for tumor molecular typing based on protein glycosylation profiles will be established. Furthermore, new solutions for sugar-targeted therapies with enhanced tissue specificity will emerge, alongside the development of predictive models for the efficacy of glyco-immunotherapy. The precision-driven diagnostic and treatment strategies for Tn/STn antigens, resulting from the synergistic intersection of glycoscience, immunology, and nanotechnology, hold the potential to drive revolutionary breakthroughs in cancer treatment.
